# Contrasting impacts of a novel specialist vector on multihost viral pathogen epidemiology in wild and managed bees

**DOI:** 10.1111/mec.15333

**Published:** 2020-01-07

**Authors:** Robyn Manley, Ben Temperton, Mike Boots, Lena Wilfert

**Affiliations:** ^1^ Centre for Ecology and Conservation University of Exeter Penryn UK; ^2^ Department of Biosciences University of Exeter Exeter UK; ^3^ Department of Integrative Biology University of California Berkeley CA USA; ^4^ Institute of Evolutionary Ecology and Conservation Genomics University of Ulm Ulm Germany

**Keywords:** epidemiology, *Varroa destructor*, viruses, wild pollinator

## Abstract

Typically, pathogens infect multiple host species. Such multihost pathogens can show considerable variation in their degree of infection and transmission specificity, which has important implications for potential disease emergence. Transmission of multihost pathogens can be driven by key host species and changes in such transmission networks can lead to disease emergence. We study two viruses that show contrasting patterns of prevalence and specificity in managed honeybees and wild bumblebees, black queen cell virus (BQCV) and slow bee paralysis virus (SBPV), in the context of the novel transmission route provided by the virus‐vectoring *Varroa destructor*. Our key result is that viral communities and RNA virus genetic variation are structured by location, not host species or *V. destructor* presence. Interspecific transmission is pervasive with the same viral variants circulating between pollinator hosts in each location; yet, we found virus‐specific host differences in prevalence and viral load. Importantly, *V. destructor* presence increases the prevalence in honeybees and, indirectly, in wild bumblebees, but in contrast to its impact on deformed wing virus (DWV), BQCV and SBPV viral loads are not increased by *Varroa* presence, and do not show genetic evidence of recent emergence. Effective control of *Varroa* in managed honeybee colonies is necessary to mitigate further disease emergence, and alleviate disease pressure on our vital wild bee populations. More generally, our results highlight the over‐riding importance of geographical location to the epidemiological outcome despite the complexity of multihost‐parasite interactions.

## 1 INTRODUCTION

The majority of pathogens exist in complex communities infecting multiple host species (Woolhouse, Taylor, & Haydon, 2001). Within such multihost systems, host species typically vary in their abundance, ecology and behaviours, as well as in their susceptibility to pathogens and subsequent transmission potential (Haydon, Cleaveland, Taylor, & Laurenson, 2002; Streicker, Fenton, & Pedersen, 2013). There are important examples, such as West Nile virus, where a key species drives a disproportional amount of the disease persistence and transmission to sympatric hosts (Kilpatrick, Daszak, Jones, Marra, & Kramer, 2006). The addition of a new transmission route, such as a vector, into these complex multihost transmission cycles can have important implications to disease dynamics, and the vector's behaviour and population dynamics may need to be taken into account (Dobson, 2004). By parasitising multiple hosts with varying transmission potential, a generalist vector can dilute the transmission potential of a key host, as demonstrated by the effect of increased mammal diversity on Lyme disease incidence: Higher mammalian host diversity reduced the disease transmission role of the white‐footed mouse, the most competent reservoir host of *Borrelia burgdorferi* (the causative agent of Lyme disease) (Kilpatrick et al., 2006), thereby reducing Lyme disease risk (LoGiudice, Ostfeld, Schmidt, & Keesing, 2003). In contrast, a specialist vector can increase the transmission potential of a key host; for example, the American Robin is believed to be responsible for the West Nile virus epidemic in New York due to preferential feeding behaviour of a mosquito vector on this relatively rare but highly competent host (Kilpatrick et al., 2006). Thus, the epidemiology of pathogens across multiple host species, may be critical in driving disease emergence, and ultimately for disease control.

Pollinators are host to a large number of RNA viruses that are known to be pathogenic to honeybees, but have more recently been identified as multihost pathogens, prevalent in wild bee populations (Evison et al., 2012; Fürst, McMahon, Osborne, Paxton, & Brown, 2014; Levitt et al., 2013; Manley, Boots, & Wilfert, 2015; Manley et al., 2019; McMahon et al., 2015; Singh et al., 2010). There are known differences in viral prevalence and viral load across honeybee and bumblebee hosts for many RNA viruses (McMahon et al., 2015). Deformed wing virus (DWV) is undergoing a global epidemic in honeybees and is an emerging disease in bumblebees (Fürst et al., 2014; Manley et al., 2019; Wilfert et al., 2016); its prevalence and viral load is highest in honeybees (*Apis mellifera*). Black queen cell virus (BQCV) is also closely linked with honeybees (McMahon et al., 2015) but with high prevalence and viral load found across bumblebee species, particularly when apiaries are present in the area (Alger, Burnham, Boncristiani, & Brody, 2019). Slow bee paralysis virus (SBPV) on the other hand shows higher prevalence and viral load in bumblebee species than in honeybees (McMahon et al., 2015). Alongside DWV, SBPV has been assigned to the genus *Iflavirus* (de Miranda et al., 2010), while BQCV is a member of the *Dicistroviridae* family. Deformed wing virus is linked to high overwinter mortality of honeybee hives and increased worker mortality in bumblebees (e.g., Berthoud, Imdorf, Haueter, Radloff, & Neumann, 2010; Dainat, Evans, Chen, Gauthier, & Neumann, 2012; Fürst et al., 2014; Genersch et al., 2010; Highfield et al., 2009; Natsopoulou et al., 2017). Slow bee paralysis virus has been shown to infect *Bombus terrestris*, significantly reducing longevity under nutritional stress (Manley et al., 2015).

The mechanisms behind DWV's host heterogeneity are relatively well understood: *A. mellifera* has been strongly implicated as the ancestral and reservoir host for DWV (Fürst et al., 2014; Wilfert et al., 2016) in association with the virus‐vectoring ectoparasitic mite, *Varroa destructor* (Manley et al., 2019; Wilfert et al., 2016). *Varroa destructor* jumped from its native Asian host, *Apis ceranae*, to the western honeybee *A. mellifera*, in the middle of last century, and has since spread worldwide (Oldroyd, 1999) causing high colony mortality as a vector of DWV (e.g., Dainat et al., 2012; Genersch, 2010; Highfield et al., 2009). Deformed wing virus potentially increases in the mite by replication (Gisder, Aumeier, & Genersch, 2009; Ryabov et al., 2014), or possibly through bioaccumulation of virus particles through blood feeding (Erban et al., 2015). While *V. destructor* is a specialist vector restricted to honeybees, it indirectly increases DWV prevalence and titre in sympatric bumblebees by dramatically increasing the transmission potential of *A. mellifera* (Manley et al., 2019), i.e., *A. mellifera* becomes a “superspreader” host species (Lloyd‐Smith, Schreiber, Kopp, & Getz, 2005; Manley et al., 2019).

RNA viruses differ in their association with *V. destructor* (hereafter referred to as *Varroa* for simplicity), which will influence the risk of virus emergence in wild bumblebees. There is currently no clear evidence associating BQCV with transmission by *Varroa* (Locke, Forsgren, Fries, & De Miranda, 2012; Ribière, Ball, & Aubert, 2008; Tentcheva et al., 2004), although one study found a weak correlation of BQCV titre with *Varroa* infestation rates in New Zealand (Mondet, De Miranda, Kretzschmar, Le Conte, & Mercer, 2014). Slow bee paralysis virus differs from this pattern: *Varroa* has been shown experimentally to be capable of transmitting SBPV (Santillán‐Galicia, Ball, Clark, & Alderson, 2014), a virus which is more prevalent in *Varroa*‐positive colonies (Carreck, Ball, & Martin, 2010). However, in the wild, SBPV has been found at higher prevalence in certain bumblebee species (specifically in *Bombus hortorum*) than in *A. mellifera* (McMahon et al., 2015), suggesting that *A. mellifera* is not the reservoir host for this virus.

*Varroa* has invaded the entire European mainland with the exception of several island refuges off the coast of the British Isles and the French coast. Using single‐molecule RNA sequencing, we take advantage of this natural experiment to examine how *Varroa* has impacted on the diversity and composition of RNA viromes across honeybee and bumblebee hosts. Further, we focus on the epidemiology of two multihost RNA viruses, SBPV and BQCV – two viruses with different apparent patterns of host specificity – to examine whether these differences affect the epidemiological history of these viruses and whether this ultimately results in different scenarios for how the acquisition of a specialist virus affects pathogen transmission.

## 2 MATERIALS AND METHODS

We collected foraging bees (355 *A. mellifera*, 281 *Bombus pascuorum*, 640 *B. terrestris* and 38 *Bombus lucorum* individuals) within a 1 × 1 km area as described in detail in (Manley et al., 2019), from four *Varroa*‐free islands; three *Varroa*‐positive islands; and five *Varroa*‐positive mainland sites (Figure S1, Table S1). We differentiated between *B. terrestris* and *B. lucorum* via a mtDNA length polymorphism (Table S2). We extracted DNA from homogenised gut tissue using Chelex following the manufacturer's instructions, and RNA using Trizol and bromo‐chloropropane from individuals (gut homogenate and half the head and thorax (bisected laterally)) as described in detail in Manley et al. (2019). RNA was resuspended in 100 μl (*A. mellifera*) or 400 μl (*Bombus* species) of nuclease‐free water. cDNA transcription was performed on 2 µl of resuspended RNA using GoScript Reverse Transcriptase (Promega), with random hexamer primers and RNasin to prevent RNA degradation. To determine viral prevalence of BQCV and SBPV we carried out PCR in 20 µl reactions using GoTaq DNA Polymerase (primers and programs in Table S2) and ran the products on 1.5% TAE agarose gel with ethidium bromide nucleic acid staining solution.

We randomly selected 10 BQCV and SBPV positives per site/species (or total number if there were fewer than 10 positives available) for qRT‐PCR analysis (BQCV: *n* = 148, SBPV: *n* = 141). We measured RNA concentration (Qbit Fluorometer) and quality (Nanodrop 2000 spectrophotometer) for all samples; all samples had a 260/280 nm ratio between 1.8 and 2.1. We performed cDNA transcription on 2 µl of 400 ng RNA template using GoScript Reverse Transcriptase and diluted the cDNA 1:10 prior to qRT‐PCR. Duplicate reactions were run for each sample on a Strategene machine (Mx3005P) using GoTaq qPCR Master mix for dye‐based detection (Promega, Table S3), alongside two no‐template negative controls. We calculated viral copy number using duplicate eight‐point standard curves of plasmid DNA, of known quantity, in a 1:10 serial dilution on each plate. We generated BQCV and SBPV plasmids using Promega pGEM‐T Easy Vector to clone a 257 bp fragment of ORF 2 of the BQCV genome and 186 bp fragment of the VP2‐gene in SBPV from purified PCR products (primer details in Table S3), selecting successful transformants via blue/white screening. Plasmids were extracted using GeneJET Plasmid Miniprep Kit (ThermoFisher). We used M13 primers (designed to sequence inserts inside pGEM‐T Easy Vector: forward 5′‐GTTTTCCCAGTCACGAC‐3′, reverse 5′‐CAGGAAACAGCTATGAC‐3′) to confirm the correct product had been cloned. We linearlised the plasmids using the restriction enzyme Apa 1 (New England Biolabs), according to the manufacturer's instructions, and diluted them 1:1,000 with nuclease‐free water. Mean efficiency across plates for BQCV was 95.7% (four plates ranging from 93.5%–96.7%) and SBPV was 95.2 (five plates ranging from 91.6–101.8) with *R* ^2^ > .98 across assays.

The same positive samples were assayed by PCR for one genomic region in BQCV and SBPV (Table S2), purified using Exonuclease 1 and Antarctic phosphatase by incubation at 37°C for 60 min and denaturation at 80°C for 20 min, and sequenced using Big Dye Terminator v3.1 (Applied Biosystems) on an ABI 3730 Genetic Analyser. Sequencing direction was chosen to optimise the number of sequences and the length of amplicon. We inspected all sequences manually in Geneious (v6.8) for quality and excluded any sequences based on the following quality criteria: heterozygosity, short sequences (<432 nt for BQCV and <535 nt for SBPV), and >3 ambiguous bp. This resulted in alignments of 432 nt for 69 individuals for BQCV (reference genomes BQCV NC003784.1) and 535 nt for 78 individuals for SBPV (reference genome GU938761.1) (Table S4). To confirm that there was no recombination within fragments at a *p*‐value of .05, we used the GENECONV (Padidam, Sawyer, & Fauquet, 1999), MaxChi (Maynard Smith, 1992), BootScan (Martin, Posada, Crandall, & Williamson, 2005) and SiScan (Gibbs, Armstrong, & Gibbs, 2000) algorithms in the rdp4 package (v4.56) (Martin, Murrell, Golden, Khoosal, & Muhire, 2015).

We ran Jmodeltest to compare and select an appropriate evolutionary substitution model for each alignment based on the Bayesian Information Criterion (Alizon & Fraser, 2013) for use in phylogenetic reconstructions (Table S4). We fit discrete trait models with asymmetric substitution models for host species and geographic location, which allows transitions to and from a host or location to occur at different rates (trait rate and indicators operators weight = 1), implemented in Beast v1.8 (Drummond & Bouckaert, 2015). We ran and compared nine models concurrently for each alignment with different demography and molecular clock rates, and used the path sampling maximum likelihood estimator, implemented in Beast 1.8, to determine the best model (Table S5). For BQCV, the preferred model used a HKY+I+G substitution model with an exponential relaxed molecular clock and a constant population size prior. For SBPV, the preferred model used a HKY+G substitution model, with a lognormal relaxed molecular clock and a constant population size prior. Models were run without prior knowledge of the evolutionary rate. Black queen cell virus models were run for 10,000,000, and SBPV models for 20,000,000 MCMC steps with sampling every 1,000 or 2,000 generations, respectively. Posterior distribution, convergence and effective sample size was assessed using Tracer v1.6 (Drummond & Rambaut, 2007): all models achieved high effective sample size (>200). We produced Maximum Clade Credibility (MCC) trees (TreeAnnotator (v1.8.4)) to infer host ancestral state probabilities. We used MrBayes 3.2.6 to produce Bayesian phylogenetic trees for each alignment. Phylogenetic tree figures were plotted using Figtree v1.4.3. Well‐supported migration rates between host species and sites were identified using Bayes factors (SPREAD v1.0.6.), with a Bayes Factor of 3 as a cutoff. For BQCV and SBPV sequence alignments (Table S4) we produced median joining phylogenetic networks using PopArt (v.1.7). We calculated Tajima's *D*, Kst (Hudson, Boos, & Kaplan, 1992) and the nearest neighbour statistic S_NN_ (Hudson, 2000) using DNASPv5.10.1 (Librardo & Rozas, 2009).

We created eight comparable populations for SMRT sequencing by pooling 1,000 ng of RNA from 30 *A. mellifera* and 30 *B. terrestris* individuals, from two *Varroa*‐free sites (Ushant and the Isle of Man) and two *Varroa*‐positive mainland sites (Le Conquet and Liverpool). Note: *B. terrestris* was rare on Ushant, thus only 13 individuals were collected and used in that pool. Pools all had high RIN values (>9) (Agilent 2200 Tapestation) except for Liverpool honeybees with RIN = 7.6. Exeter Sequencing Service prepared full‐length cDNA libraries using the BluePippin System, followed by Clontech SMARTer PCR cDNA Synthesis Kit and generated SMRTbell libraries using the PacBio Template Prep Kit, which were then sequenced on the PacBio System. To first remove host‐derived sequences, we mapped nonchimeric reads from each pool against their respective host species genomes using BWA (Li & Durbin, 2009) (v. 0.7.12) with the following parameters: “bwa mem ‐x pacbio.” We then mapped remaining reads against all sequenced RNA viruses and 23 novel bumblebee viruses (Pascall, Tinsley, Obbard, & Wilfert, 2018) (Table S6). We ran principle component analysis (PCA) in R (v3.6.1) using the prcomp function to look at similarity in viral composition between pools. We calculated traditional measures of diversity for each pool: Simpson's Diversity Index (1‐*D*) and Shannon's Diversity Index (*H*). Reads that did not map to either known viruses or hosts were assembled with canu (v.1.5) to look for novel viruses with the following parameters: ‘genomeSize=100k useGrxml:id=false ‐pacbio‐raw contigFilter**=**“2 1000 1.0 1.0 2”. Assemblies were analysed using the gene annotator software genemark (http://opal.biology.gatech.edu/GeneMark), with each putative gene examined individually using BLASTP+.

We excluded *B. lucorum* samples (*n* = 38) and the single *B. pascuorum* from Quiberon from prevalence analyses because of low sample size. We used RStudio (v0.99.896) for all statistical analyses. To account for imperfect detection of viruses by PCR assays, we calculated true prevalence with 95% confidence intervals (using R library epiR v0.9‐82 and the function epi.prev, with the confidence level conservatively set at 95% [Reiczigel, Foldi, & Ozsvari, 2010] and confidence intervals calculated based on methods in Blaker (2000)). To test if BQCV and SBPV prevalence was affected by *Varroa*‐presence we ran generalised linear mixed models (GLMMs) lme4 package (v1.1‐12) (Bates, Maechler, Bolker, & Walker, 2015) with binomial error distribution and logit link function. In the full models we included three‐way interaction between fixed effects; *Varroa*‐presence/absence, species (a factor with three levels: *A. mellifera*, *B. terrestris* and *B. pascuorum*) and island/mainland location; latitude and sunshine hours duration were included as proxies for favourable disease transmission as additional fixed effects (Fürst et al., 2014; Manley et al., 2019): field site and individual were included as random effects (individual was added to account for over‐dispersion in the models [Harrison, 2014]). We removed nonsignificant terms using the ‘ANOVA’ function to determine the minimum adequate model (MAM). We compared the MAM with the null model (which only included random effects) using ANOVA to test the full effect of our predictors and examined residual plots to assess model fit. We ran GLMMs to examine if viral load was affected by *Varroa*‐presence: BQCV and SBPV were tested in separate models with Gamma error distribution and inverse link function. Viral load data were log transformed before analysis because the data varied across orders of magnitude from 10^3^ to 10^10^.

We sampled on *Varroa*‐ positive islands, as well as paired *Varroa*‐ positive mainland sites, to test for a possible island effect on disease prevalence. Further, we ran models on reduced data sets to rule out the effect of island location on disease prevalence (a) comparing island sites with and without *Varroa*, and (b) comparing *Varroa*‐positive islands and mainland sites.

To assess if coinfection of BQCV and SBPV, as well as DWV‐A and DWV‐B (Manley et al., 2019), occurred more often than expected (based on the individual prevalence rate), we used Chi‐square pairwise comparisons with Bonferroni corrected *p‐*values and Yates' continuity correction. We ran test of proportions to investigate if *Varroa*‐presence affects rates of coinfection.

## 3 RESULTS

### 3.1 Virome diversity

**Figure 1:**
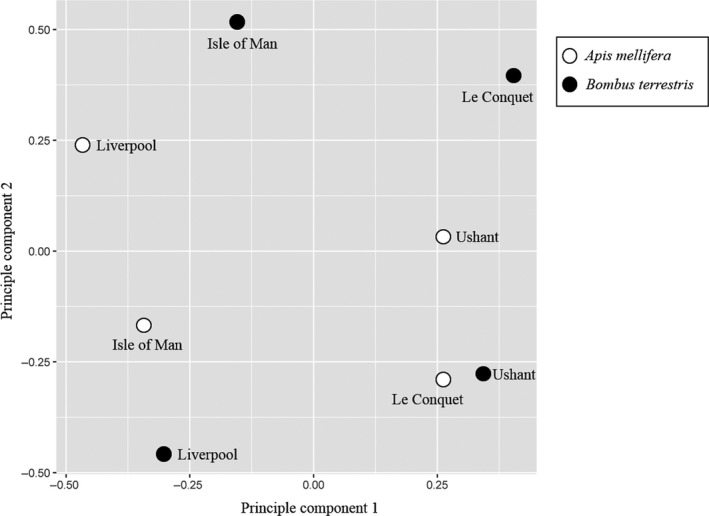
Principle component analysis on the viral composition of eight bee populations, based on single molecule real‐time (SMRT) sequences mapped by BWA‐mem to all previously sequenced “honeybee” viruses and fragments from 23 newly discovered bumblebee viruses (Table S6). *Apis mellifera* populations are shown with white circles and *Bombus terrestris* populations shown with black circles. 38.8% of the variance is explained by PC1, and the cumulative % variance explained by PC1 and PC2 is 62.7%

PacBio single molecule RNAseq data suggest that geographic location, rather than host species or *Varroa* presence/absence, determines the overall viral composition of pollinator populations as well as the viral diversity. A principal component analysis of viral PacBio reads (Figure 1) shows that populations from Liverpool and the Isle of Man (located in the North of England and the Irish Sea, respectively; see Figure S1) are clearly separated from Le Conquet and Ushant (located in Brittany and off the Breton coast, respectively) along the first principal component (38.8% of variance). Both *A. mellifera* and *B. terrestris* populations from Liverpool and the Isle of Man carried a more diverse viral fauna (mean Simpson's Index of Diversity [1‐*D*] = 2.33 [range: 1.91–2.62] and mean Shannon's diversity Index [*H*] = 1.17 [range: 0.92–1.36]) than populations from Le Conquet and Ushant (1‐*D* = 1.31 [range: 1.02–2.00], *H* = 0.31 [0.05–0.72] two‐sample *t* tests: Simpson's index: *t* = 4.03 [*df* = 5], *p* = .016; Shannon's index: *t* = 4.03 [*df* = 5], *p* = .005). *Varroa* presence has no effect on viral diversity (two‐sample *t* test of viral diversity at *Varroa‐*present sites compared to *Varroa‐*free sites: Simpson's index: *t* = 3.71 [*df* = 6], *p* = .50; Shannon's index: *t* = 4.03 [*df* = 6], *p* = .50). While total read numbers cannot be used quantitatively because of amplification steps during library preparation, relative read numbers of each virus are comparable between samples. The total number of virus reads from the *B. terrestris* populations are significantly lower compared to reads from *A. mellifera* populations from the same location (tests of proportions: Brest χ^2^ = 165,451, *p* < .001; Liverpool ‐ χ^2^ = 6761, *p* < .001; Isle of Man ‐ χ^2^ = 2,8431, *p* < .001; Ushant was the exception with significantly more reads in *B. terrestris* compared to *A. mellifera* [χ^2^ = 841, *p* < .001]).

A striking finding was that DWV‐B is the dominant virus across *A. mellifera* and *B. terrestris* populations from all sites, comprising 75% of all virus reads, while DWV‐A is rare, comprising only 0.07% of reads (Table S7). *Apis mellifera* filamentous virus (Amfv), a double‐stranded DNA virus distantly related to Bracoviruses (Gauthier et al., 2015), is also present in all populations across host and site (6.8% of reads), although highest in *A. mellifera* populations from Ushant and Isle of Man and relatively rare in *B. terrestris* populations (Table S7). Slow bee paralysis virus (Harpenden and Rothamsted strains) (12.9% of reads), Sacbrood virus (4.4% of reads) and BQCV (0.5% of reads) are notably common in the Liverpool/Isle of Man populations but mostly absent from the Le Conquet/Ushant populations (for map of sites see Figure S1); reads from all other viruses (Table S6) are rare (including four newly discovered bumblebee viruses [Pascall et al., 2018]), or absent across all populations. We found no evidence for further novel viruses within these pools from analysis of contigs assembled from reads that did not map to either host or viral genomes.

### 3.2 Prevalence and host specificity of BQCV and SBPV

**Figure 2:**
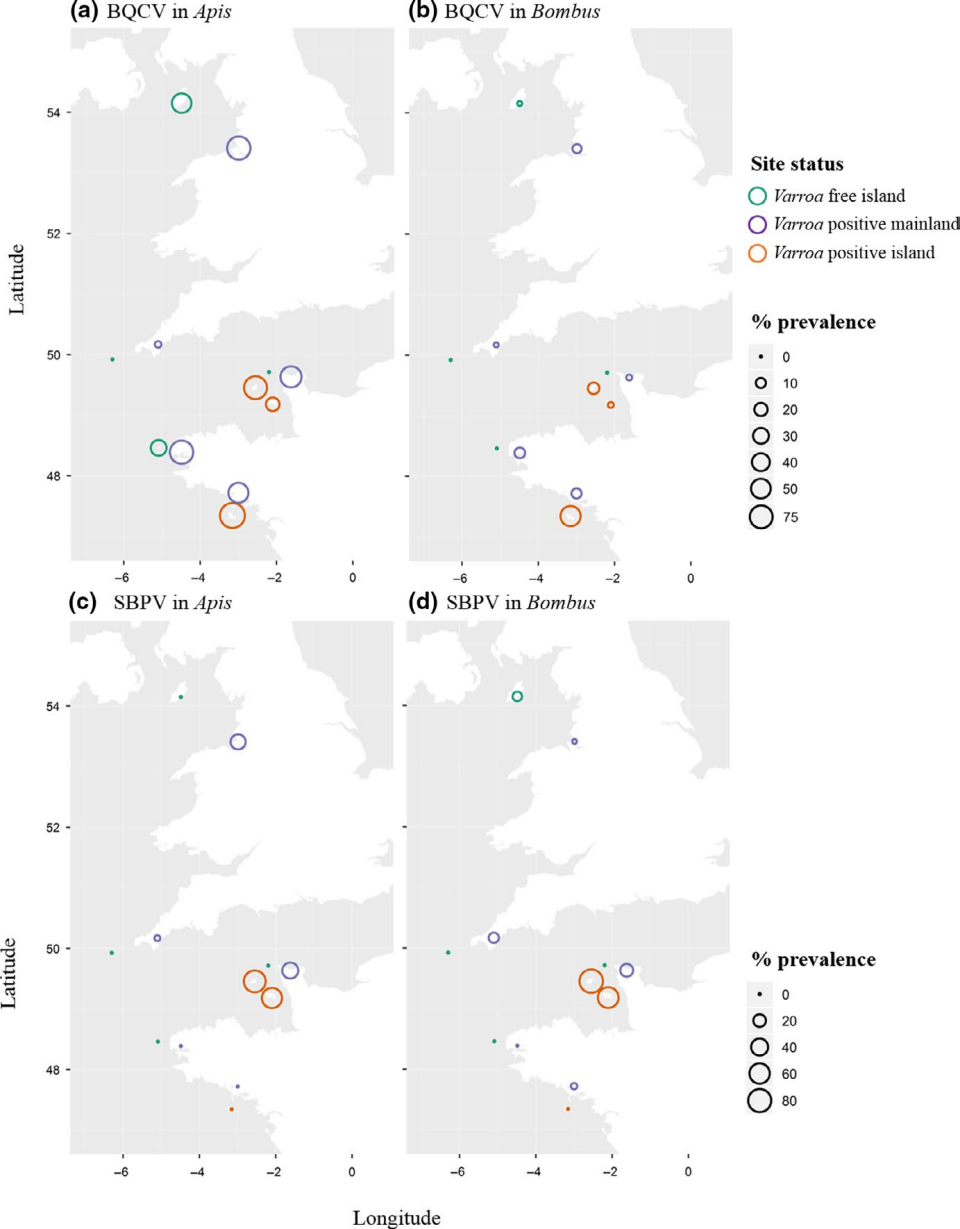
Individual prevalence of Black queen cell virus (BQCV) in (a) *Apis mellifera* and (b) *Bombus* spp. and (c) Slow bee paralysis virus (SBPV) in *Apis mellifera* and (d) *Bombus* spp. mapped across 12 field sites. Scales represent % prevalence and are different for each virus [Colour figure can be viewed at http://www.wileyonlinelibrary.com]

Prevalence screens across our 12 populations show that both BQCV and SBPV are found in honeybees and bumblebees (Figure 2). Black queen cell virus is more prevalent in *A. mellifera,* three times and >20 times more prevalent compared to *B. terrestris* and *B. pascuorum*, respectively (tests of proportions between *A. mellifera* and *Bombus* spp. combined: BQCV χ^2^ = 229.75_1_, *p* < .001) (Table 1). Prevalence of SBPV, in contrast to the other viruses, was highest in *B. pascuorum*, being 3× higher compared to *B. terrestris* (χ^2^ = 29.86_1_, *p* < .001) and 1.5× higher compared to *A. mellifera* (χ^2^ = 4.89_1_, *p* = .027) (Table 1).

**Table 1:** True prevalence (with 95% confidence intervals) of Black queen cell virus (BQCV) and Slow bee paralysis virus (SBPV) detected using PCR (primer details Table S1)

	BQCV	SBPV
*Apis mellifera* (*N* = 355)	46.4 (40.58–52.2)	16.97 (12.49–21.92)
*Bombus terrestris* (*N* = 640)	7.81 (5.14–10.88)	8.85 (6.09–12.01)
*Bombus pascuorum* (*N* = 280)	2.14^a^ (0.9–4.5)	25.79 (20.13–32.08)

a For populations where true prevalence = 0 (because it is based on 95% sensitivity and specificity of the PCR assay) we report actual prevalence based on our data (positives confirmed by Sanger sequencing).

**Figure 3:**
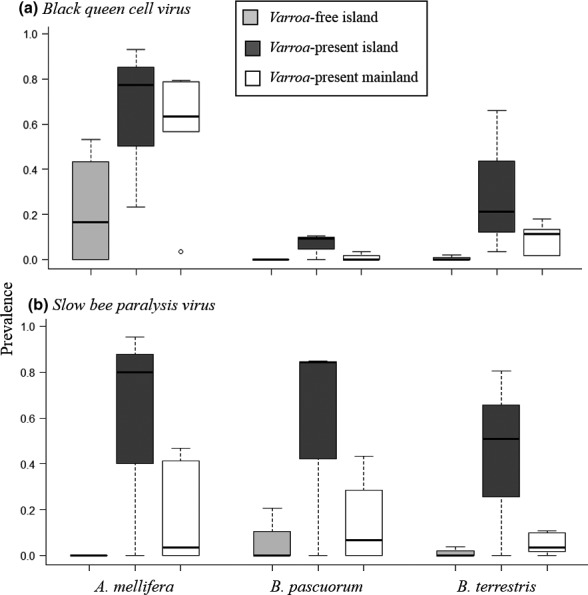
Prevalence of Black queen cell virus (BQCV) and Slow bee paralysis virus (SBPV) across host species and *Varroa*‐presence: *Varroa*‐free sites (V−, light grey), *Varroa*‐present islands (V + I, dark grey), and *Varroa‐*present mainland (V + M, white)

In GLMMs, host species was a significant predictor of BQCV prevalence, but not for SBPV (Table 2), and was thus removed from the model by model selection (SBPV χ^2^ = 2.42 (*df* = 2), *p* = .30). *Varroa* presence was a significant predictor for BQCV and SBPV prevalence (Table 2, Figure 3). *Varroa* presence predicts a 10‐fold increase in BQCV prevalence in *A. mellifera* and a 20‐fold increase in both *B. terrestris* and *B. pascuorum*; and an increase in SBPV prevalence across host species by over 100‐fold (Table S8). The influence of *Varroa* on viral prevalence is highlighted by the apparent absence of viruses in two of the four *Varroa*‐free sites (Scilly Isles and Alderney) (Figure 2). Interactions between *Varroa* presence and host species were included in each full model, but were removed by model selection (ANOVA: BQCV χ^2^ = 2.47 [*df* = 2], *p* = .30; SBPV χ^2^ = 5.85 [*df* = 2], *p* = .054) indicating that *Varroa* presence increases prevalence in both honeybees and bumblebees equally. Sunshine hours and latitude served as proxies for favourable conditions for disease transmission, sunshine hours were not significant and removed from all models by model selection (BQCV χ^2^ = 0.37 [*df* = 1], *p* = .54, SBPV χ^2^ = 0.25 [*df* = 1], *p* = .62), while latitude had no effect on BQCV prevalence (BQCV χ^2^ = 0.16 [*df* = 1], *p* = .70), but could not be excluded from SBPV prevalence models (χ^2^ = 6.05 [*df* = 1], *p* = .02) (Table 2). Island‐mainland location was also included as a fixed factor and excluded by model selection for both viruses (ANOVA: BQCV χ^2^ = 0.84, [*df* = 1], *p* = .36; SBPV χ^2^ = 3.34, [*df* = 1], *p* = .07), indicating that we are indeed seeing an effect of *Varroa* presence, rather than an island effect. Our full models for viral prevalence fitted the data significantly better than the null model that contained random factors only (ANOVA: BQCV χ^2^ = 46.64 [*df* = 3], *p* < .001, SBPV χ^2^ = 7.75 [*df* = 3], *p* = .02).

**Table 2:** GLMMs with binomial error structure and logit function: Black queen cell virus (BQCV) and Slow bee paralysis virus (SBPV) prevalence as a response to *Varroa* presence and host species (minimum adequate models)

Pathogen prevalence	Parameters	Estimate	*SE*	*z*‐value	*p*‐value
BQCV	Intercept	−3.75	1.67	−2.24	.025
	*Bombus pascuorum*	−4.93	0.53	−9.30	<.001
	*Bombus terrestris*	−2.96	0.28	−10.54	<.001
	*Varroa* presence	3.83	1.47	2.62	.009
SBPV	Intercept	−7.74	2.22	−3.46	<.001
	Latitude (log)	116.72	66.47	1.76	.022
	*Varroa* presence	4.98	2.44	2.05	.041

To further confirm that *Varroa* presence, rather than an island effect, explained viral prevalence, we ran GLMMs on a reduced data set excluding *Varroa‐*free sites (i.e., comparing three *Varroa‐*present islands with five *Varroa‐*present mainland sites) and with island as an explanatory variable: there was no significant difference in BQCV or SBPV prevalence on *Varroa*‐present islands compared to *Varroa*‐present mainland (estimate ± *SE* of the fixed factor “island/mainland” in the model = 1.17 ± 1.10, *p* = .28; 2.3 ± 2.1, *p* = .26, respectively). In addition, we ran the same models on a data set excluding mainland sites (i.e., comparing four *Varroa‐*free islands to three *Varroa‐*present islands), here *Varroa* presence remained a significant explanatory factor of BQCV and SBPV prevalence, with higher prevalence on *Varroa‐*present islands compared to *Varroa*‐free islands (estimate ± *SE* of the fixed factor *Varroa* presence in the model = 3.76 ± 1.49, *p* = .018; 7.19 ± 3.87, *p* = .063, respectively). It is notable that SBPV was found at extremely high prevalence on two *Varroa*‐present islands, Guernsey and Jersey, but is absent on Belle‐Ile (the third *Varroa*‐present island). However, prevalence is also high and absent on the closest mainland sites, Cherbourg and Quiberon, respectively, indicating a location difference rather than an island effect.

### 3.3 Virus transmission potential

**Figure 4:**
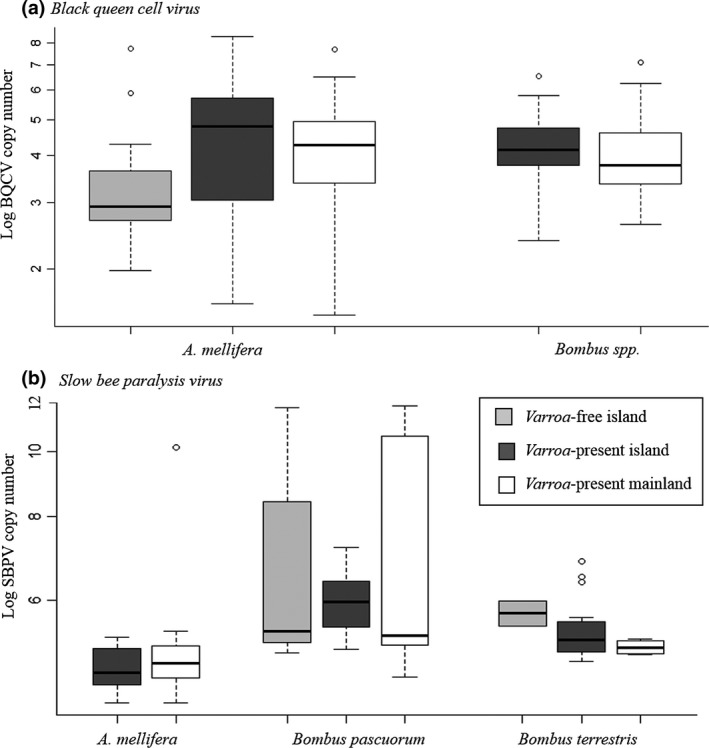
Log viral copy number of Black queen cell virus (BQCV) and Slow bee paralysis virus (SBPV) across host species (note, only genus is shown for BQCV as there was no significant difference between host) and *Varroa*‐presence: *Varroa*‐free sites (V−, light grey), *Varroa*‐present islands (V + I, dark grey), and *Varroa‐*present mainland (V + M, white)

Viral loads ranging from 10^3^–10^10^ were found for both viruses (Figure 4). Host species is a significant factor predicting SBPV viral load (GLMM results reported in Table 3). *Bombus pascuorum* has significantly higher SBPV loads than *A. mellifera* (Figure 4, KS‐test: *D* = 0.71, *p* < .001), with the GLMM predicting SBPV loads in *B. pascuorum* to be one order of magnitude higher than in *A. mellifera* and *B. terrestris*. There were no significant differences in viral load between host species for BQCV (KS‐test: *D* = 0.18, *p* = .37, GLMM results in Table 3). Although *Varroa* influences prevalence of both BQCV and SBPV (Table 2, Figure 3), it is not a significant predictor of viral load (Table 3, Figure 4). Latitude, duration of sunshine hours and island/mainland had no influence on viral load and were removed from the models by model selection.

**Table 3:** GLMMs on Slow bee paralysis virus (SBPV) viral load with Gamma error structure

Pathogen viral load	Parameters	Estimate	*SE*	*z*‐value	*p*‐value
SBPV	Intercept	0.20	0.024	8.04	<.001
	*Bombus pascuorum*	−0.05	0.013	−3.45	<.001
	*Bombus terrestris*	−0.016	0.014	−1.18	.24
	*Varroa* present	0.004	0.023	0.20	.84
BQCV	Intercept	0.27	0.025	10.80	<.001
	*Bombus pascuorum*	−0.03	0.040	−0.84	.40
	*Bombus terrestris*	0.02	0.020	1.33	.18
	*Varroa* present	−0.04	0.053	−0.70	.48

### 3.4 Coinfection of viruses

Coinfection occurred in all combinations between BQCV and SBPV, as well as DWV‐A and DWV‐B (Manley et al., 2019). Coinfection was rare in bumblebees and common in *A. mellifera*, but only in *Varroa*‐positive sites (Figure S2). True prevalence of coinfection of two viruses in *Apis* across all sites was 25.4% (95% C.I. 20.3–30.9) and in *Bombus* was 2.4% (C.I. 0.7–4.8); coinfection of three and four viruses was rare (true prevalence for coinfection of three viruses was 9.7% in *Apis* (C.I. 6.1–14.2) and <1% in *Bombus*, and only 11 *A. mellifera* were coinfected with four viruses. Coinfection was significantly more likely in *Varroa‐*positive sites for all hosts (test of proportions: *A. mellifera* χ^2^ = 55.98_2_, *p* < .001; *B. terrestris* χ^2^ = 89.05, *p* < .001; *B. pascuorum* χ^2^ = 12.82, *p* = .002), as expected given the higher viral prevalence in these sites. The proportion of coinfected individuals was higher than expected based on single infection rates (χ^2^ = 150.33_3_, *p* < .001). Further, pairwise comparisons of the four viruses, using Bonferroni corrected *p*‐values (*p* = .008) and Yates' continuity correction, revealed that DWV‐A presence was linked to DWV‐B, BQCV and SBPV presence (χ^2^ = 142.18_1_, *p* < .001, χ^2^ = 102.3_1_, *p* < .001 and χ^2^ = 106.96_1_, *p* < .001, respectively). Indeed, DWV‐A only occurred in coinfection with DWV‐B. In contrast, DWV‐B, BQCV and SBPV presence were all independent of each other. We confirmed these results in an analysis excluding the DWV‐A *Varroa* negative sites. Further, we carried out the same analysis on *Varroa*‐free sites in the absence of DWV‐A, SBPV presence was linked to DWV‐B and BQCV presence (χ^2^ = 7.69_1_, *p* < .005; χ^2^ = 12.02_1_, *p* < .001, respectively). BQCV and DWV‐B presence remained independent of each other.

### 3.5 Viral population genetics

SBPV and BQCV populations are highly structured by geographic location, not host species. Kst values for genetic differences between locations are significant (note: Kst and Snn values close to 1 represent strong population differentiation [Hudson et al., 1992; Hudson, 2000]); Kst_SBPV_ = 0.78, *p* < .001 and Kst_BQCV_ = 0.79, *p* < .001. In addition, samples that are genetic nearest neighbours largely come from the same populations: Snn_SBPV_ = 0.97, *p* < .001, Snn_BQCV_ = 0.93, *p* < .001. SBPV and BQCV show some weak host differentiation on the edge of significance (Kst_SBPV_ = 0.034, *p* = .040, Kst_BQCV_ = 0.019, *p* = .068).

**Figure 5:**
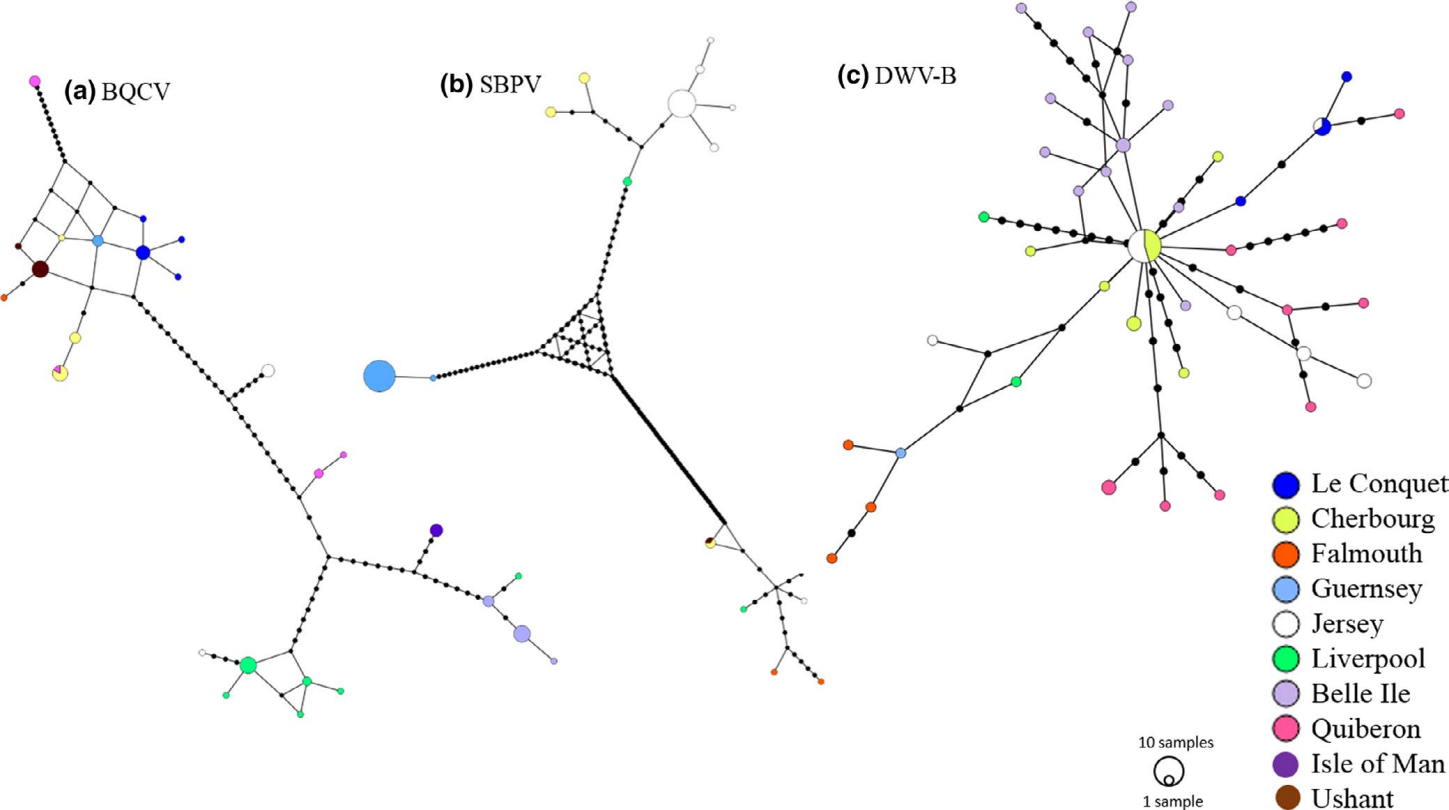
Median joining phylogenetic network of sequences from two viruses (a) Black queen cell virus (BQCV) (*N* = 69), (b) Slow bee paralysis virus (SBPV) (*N* = 78) and (c) DWV‐B (reproduced from Manley et al., 2019). The colours represent sampling location, the size of the node represents the number of samples with the same sequence and dashes on branches show the number of mutations between nodes [Colour figure can be viewed at http://www.wileyonlinelibrary.com]

**Figure 6:**
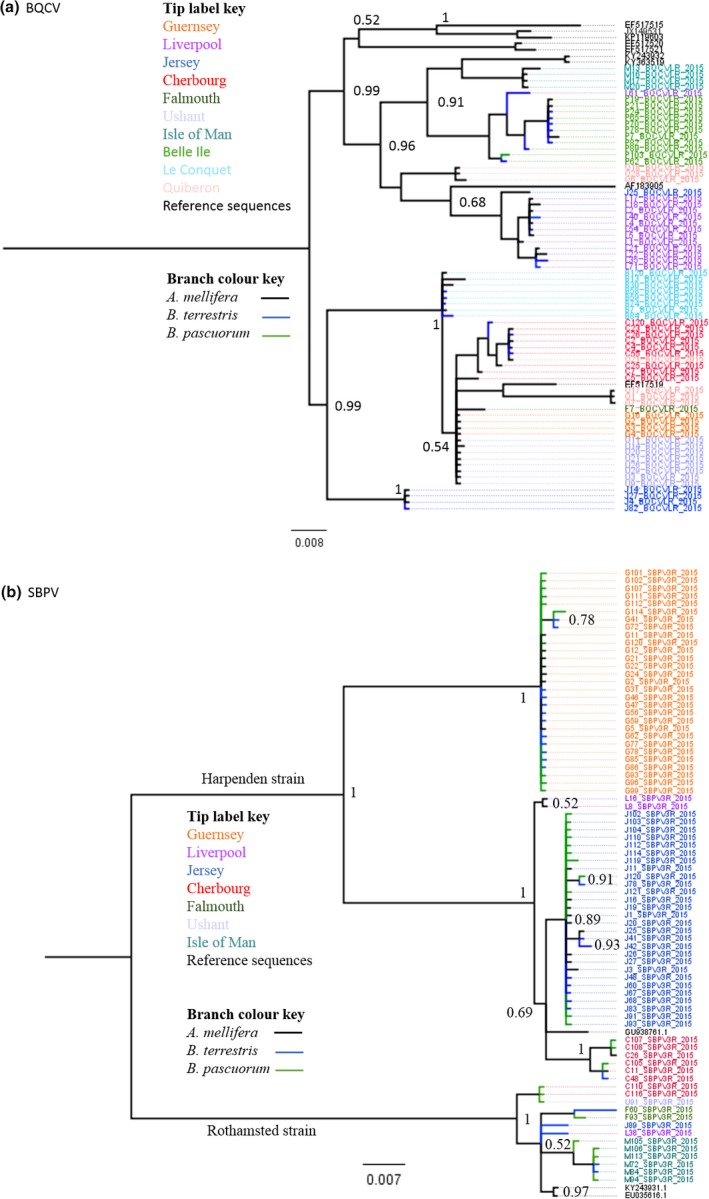
MrBayes phylogenetic tree for Black queen cell virus (BQCV) (a) A total of 69 sequences of a 432 nt fragment plus eight Genbank sequences, all isolated from *Apis mellifera,* and Slow bee paralysis virus (SBPV) (b) a total of 78 sequences of a 535 nt fragment plus three genbank sequences all isolated from *A. mellifera:* GU938761.1 (Harpenden strain), KY243931.1 and EU035616.1 (Rothamsted strain), showing posterior support (>0.5) for each node until the third order. The tip label colours represent location, branch colours represent species (see key). Samples include all sequenced isolates from the current study and for BQCV (a) eight Genbank sequences, all isolated from *A. mellifera* and for SBPV (b) three genbank sequences all isolated from *A. mellifera:* GU938761.1 (Harpenden strain), KY243931.1 and EU035616.1 (Rothamsted strain) [Colour figure can be viewed at http://www.wileyonlinelibrary.com]

Median joining phylogenetic networks for both BQCV and SBPV populations illustrate the strong geographic structuring (Figure 5a,b, respectively): BQCV (π = 0.044, with 64 polymorphic sites out of 432 examined over 69 sequences) and SBPV (π = 0.074, with 118 polymorphic sites out of 535 examined over 78 sequences). As Tajima's *D* statistic is sensitive to population structure, we restricted our analyses of demography to populations within locations, choosing those with the largest sample size. For SBPV, we examined Jersey (*N* = 28) and Guernsey (*N* = 29), and for BQCV we examined Liverpool (*N* = 13) and Belle Ile (*N* = 11), and found no evidence for an excess of rare variants (Tajima's *D* = 1.58, 1.15, −1.054, −0.3219, respectively, *p* > .05). In addition, models of exponential growth for both viruses were rejected in a Beast analysis, as the 95% HPD of the growth rate overlapped zero and a model with a constant population size prior had greater support in a path sampling analysis. Phylogenetic trees constructed using MrBayes also show strong posterior support for geographic structuring for both viruses (Figure 6a,b).

The BQCV ancestral host was identified as *A. mellifera* (state probability, *p* = 94%), while the SBPV ancestral host was identified as *B. pascuorum* (state probability, *p* = 47%). For BQCV there is support for transmission between *A. mellifera* and *B. pascuorum* (BF = 7.91) and for transmission between *B. terrestris* and *B. pascuorum* (BF = 4.9) (Table S9a). For SBPV, there is strong support for transmission between all species, but strongest support for transmission between *A. mellifera* and *B. pascuorum*, and vice‐versa (BF = 30.14, BF = 28.78, respectively, Table S10a). There is no clear pattern of transmission routes between locations; for example, locations nearest each other did not have the strongest support for state transition rates (Tables S9b and S10b, Figure S3; MCC trees for BQCV and SBPV see Figure S4a,b).

## 4 DISCUSSION

We showed that virus community composition and diversity in honeybees and bumblebees are determined by geographic location, not host species, demonstrating that shared multihost pathogens are a key feature of the pollinator community virome. While the presence of the honeybee viral vector *Varroa* does not impact overall viral community composition or diversity, it does increase viral prevalence. Furthermore, despite *Varroa* being a specialist parasite of honeybees, its presence increases the prevalence of both BQCV and SBPV not only in honeybees, but also in sympatric bumblebees. We found that *A. mellifera* and *Bombus* hosts share the same viruses and viral variants, indicating that host barriers are not a limiting factor to virus spillover and cross‐species transmission is pervasive. In contrast to the re‐emerging and strongly *Varroa*‐associated virus DWV (Manley et al., 2019), SBPV and BQCV show stable populations with strong population structure and, while viral prevalence is increased in the presence of *Varroa*, viral load, i.e., infection strength, is not affected.

We found that the overall viral community composition in *B. terrestris* and *A. mellifera* is primarily structured geographically, rather than by host species, with *B. terrestris* harbouring significantly lower read levels across all viruses detected by SMRT sequencing. Further, focusing on individual viruses, we found the same viral variants of BQCV and SBPV circulating within sympatric *A. mellifera*, *B. terrestris* and *B. pascuorum* individuals within each location. These findings strongly suggest that these viruses are continually transmitted interspecifically across sympatric species. However, prevalence of viruses significantly varies across species and viral pathogen. *A. mellifera* has previously been implicated as the reservoir host for DWV‐A and DWV‐B (Fürst et al., 2014; Manley et al., 2019), and results of the present study suggest *A. mellifera* are also the reservoir host for BQCV, inferred from both prevalence data and phylogenetic analysis: BQCV is significantly more prevalent in *A. mellifera* compared to both *Bombus* species, with *A. mellifera* identified as the ancestral host. Conversely, we identified *B. pascuorum* as the ancestral host for SBPV and record the highest prevalence and titre of SBPV in *B. pascuorum*, compared to *A. mellifera* and *B. terrestris*. These differences indicate the existence of virus‐specific host barriers to infection. Differences in host tolerance and susceptibility to each virus could also play a role in prevalence pattern. The significantly lower prevalence of DWV and BQCV in bumblebee species compared to *A. mellifera* suggests that bumblebees are poorer hosts for these viruses. Our results match the general pattern in UK bee communities, with BQCV showing highest prevalence in *A. mellifera*, but similar viral loads in honeybees and the wild bumblebee community, and SBPV with highest titre and prevalence in wild bumblebees (*B. hortorum*) (McMahon et al., 2015). There is debate about when the presence of such suboptimal hosts in a multihost system can have a dilution effect on disease transmission; or conversely, amplify transmission by increasing the abundance of both infected and susceptible hosts (Dobson, 2004; Keesing, Holt, & Ostfeld, 2006). While it is known that SBPV and DWV affect worker mortality in *B. terrestris*, such virulence data is lacking for *B. pascuorum* as well as for BQCV. To assess the potential impact of these pathogens on pollinator health, it will be essential to experimentally measure virulence in a representative range of insect pollinators. It is important to note that *Crithidia bombi*, which shows relatively small condition‐dependent effects on worker mortality (Brown, Loosli, & Schmid‐Hempel, 2000), reduces the fitness of eusocial bumblebees by up to 40% when examining this trait at a colony‐level (Brown, Schmid‐Hempel, & Schmid‐Hempel, 2003; Yourth, Brown, & Schmid‐Hempel, 2008). Pathogens may thus pose an important threat to pollinator health.

The presence of *Varroa*, a specialist vector of honeybees that is able to transmit viruses within *A. mellifera* populations, which can spill over to sympatric wild bumblebee populations via shared floral food resources (Durrer & Schmid‐Hempel, 1994; Graystock, Goulson, & Hughes, 2015), has been shown to impact multihost DWV transmission (Manley et al., 2019). We showed that while not impacting on viral composition, the presence of *Varroa* also significantly increases prevalence of BQCV and SBPV (Manley et al., 2019). Notably, we demonstrate that the presence of *Varroa* mites in honeybee populations' drive an increase in prevalence of BQCV and SBPV in honeybees, and indirectly, sympatric bumblebee hosts. The significant effect of *Varroa* on BQCV was unexpected because there is no evidence of *Varroa*‐transmission or replication within *Varroa* for this virus, with only one study correlating BQCV viral load to *Varroa* infestation (Mondet et al., 2014). Further, a previous study found no effect of *Varroa* on SBPV prevalence (Martin et al., 2012), despite associations with *Varroa* transmission (Carreck et al., 2010; Santillán‐Galicia et al., 2014). Our results suggest that the role of *Varroa* in the epidemiology of these viruses needs to be reassessed.

Crucially, BQCV and SBPV viral load is not increased by *Varroa* presence, suggesting that *Varroa* does not increase the initial titre by replication or bioaccumulation; rather these results suggest that *Varroa* can passively vector the virus between individual *A. mellifera*. One possible explanation is that the natural titre is high enough to cause maximum effect without amplification by *Varroa*, but this is unlikely given the broad range of viral titres found across naturally infected individuals (10^3^–10^11^ viral particles). It is possible that the indirect effects of *Varroa* infection such as immunosuppression (Nazzi et al., 2012) or simply the physiological damage from piercing the cuticula (Bowen‐Walker, Martin, & Gunn, 1999) could make *A. mellifera* more prone to infection with other viruses. Moreover, the well documented pathogenic association of *Varroa* with DWV infection (e.g., Bowen‐Walker et al., 1999; Evans & Schwarz, 2011; Highfield et al., 2009; Martin et al., 2012) could lead to opportunistic coinfection of *A. mellifera* by other viruses. Indeed, we found that *Varroa* presence increased the prevalence of coinfections, and that DWV‐A coinfection with DWV‐B, BQCV and SBPV occurred significantly more often than expected, suggesting that DWV‐A infection increases the chances of infection with another virus by some mechanism.

In contrast to the low genetic variation and exponential expansion of DWV‐B (Manley et al., 2019), BQCV and SBPV have higher genetic diversity, are highly structured by location, and show no exponential growth. This is further confirmed by the stark difference in pattern of the median joining phylogenetic networks (Figure 5); DWV‐B sequences form a star‐shaped network, indicating a rapidly expanding population, while both BQCV and SBPV show no evidence of population expansion, rather the patterns indicates stable populations within each location. Further, analysis restricted to populations from specific locations also showed no localised expansion for either virus. All evidence combined suggests that SBPV and BQCV are relatively stable infections compared to DWV‐B, which has recently emerged and is undergoing epidemic growth (Manley et al., 2019). There is no clear pattern of transmission routes between locations, for example locations nearest each other did not have the strongest support for routes of transmission, which gives additional support to the hypothesis that these are long term stable virus populations with strong geographic structure, obscuring any pattern of geographic movement.

In combination, our results suggest that *Varroa* presence increases intraspecific BQCV and SBPV prevalence in *A. mellifera* by direct but passive transmission, and possibly opportunistic infection of individuals weakened by *Varroa* or *Varroa‐*DWV infection: This increases spillover to wild bumblebees, resulting in increased prevalence across hosts, but has not caused epidemic outbreaks of these two viruses. This is in stark contrast to DWV‐B, where *Varroa* dramatically increases DWV prevalence and viral loads in *A. mellifera*, leading to increased spillover of high viral loads to competent bumblebee hosts, resulting in an ongoing epidemic across hosts (Manley et al., 2019). It is clear that *Varroa* plays a complex role in facilitating disease emergence in wild bumblebees. Controlling the *Varroa* mite infection in managed honeybees is vital to prevent further impact of viral disease in wild bees, already under pressure from habitat loss and pesticide use. These results demonstrate how a specialist vector can increase transmission potential for different multihost‐pathogens; the ultimate outcome – a global epidemic and disease emergence for DWV (Fürst et al., 2014; Wilfert et al., 2016) versus increased viral prevalence without increased pathogen load or epidemic growth – may depend on the specifics of host‐pathogen interactions.

## AUTHOR CONTRIBUTIONS

The study was designed by L.W., and R.M., with input from M.B. R.M. performed field and laboratory research. B.T. contributed bioinformatics analysis of SMRT data. R.M. analysed the data with input from L.W. The paper was written jointly by R.M., and L.W., with input from all authors.

## Data Availability

BQCV and SBPV sequences have been uploaded to Genbank under embargo, with accession numbers MG265504–MG265572 and MG265573–MG2656050, respectively, and will be released on publication. SMRT reads have been archived in NCBI's Sequence Read Archive with BioProject accession number PRJNA542789. Virus prevalence and qPCR data can be found on Dryad using this link: https://doi.org/10.5061/dryad.h18931zg6.
