# Supplementary material for: Contrasting impacts of a novel specialist vector on multihost viral pathogen epidemiology in wild and managed bees
Source: Mol Ecol. 2020 Jan 7;29(2):380–93. doi: 10.1111/mec.15333 (PMC7003859; doi:10.1111/mec.15333)
Supplement: Supplementary file 1 [file MEC-29-380-s001.pdf]

Supplementary material for:

Contrasting impacts of a novel specialist vector on multi-host viral pathogen epidemiology in wild and managed bees

Running title: Contrasting epidemiology of multi-host viruses

Authors: Manley, R.*[1, 2], Temperton, B.[2], Boots, M.[3] and Wilfert, L.[1,4]

Affiliations:

1. Centre for Ecology and Conservation, University of Exeter, Penryn Campus, Penryn TR11 9FE, UK
2. Department of Biosciences, University of Exeter, Streatham Campus, Exeter, EX4 4QD, UK
3. Department of Integrative Biology, University of California, Berkeley, CA, 94720, USA.
4. Institute of Evolutionary Ecology and Conservation Genomics, University of Ulm, D-89069 Ulm, Germany.

*Materials & Correspondence to:

Robyn Manley

Address: Department of Biosciences, GP309, University of Exeter, Streatham Campus, Exeter, EX4 4QD, UK.

Telephone: 01392 725 296

Email: r.manley@exeter.ac.uk

Supplementary tables

Table S1. The town and location of field site, and the total number of each bee species collected from each site. Note. *Bombus terrestris* and *B. lucorum* are cryptic and required molecular identification post-collection

Location	*Varroa*-status	*A. mellifera*	*B. terrestris*	*B. lucorum*	*B. pascuorum*
Guernsey (St Peter Port)	*Varroa*-present-islands	22	45	15	32
Jersey (St Helier)		30	59	1	33
Belle-Ile (Le Palais)		29	59	1	19
Alderney (St Anne)	*Varroa*-free-islands	30	57	3	30
Isles of Scilly (St Mary's)		30	60	0	0
Ile d'Oeussant		30	13	2	29
Isle of Man (Douglas)		32	53	5	29
Quiberon, Brittany	*Varroa*-present-mainland	30	59	0	1
Cherbourg (marina area)		30	61	3	30
Penryn (University campus)		30	56	5	30
Le Conquet, Brittany		33	59	2	19
Liverpool, city centre		29	59	0	29

Table S2. Primers and protocols to differentiate between *Bombus terrestris/lucorum* species complex, and detect prevalence of Black queen cell virus (BQCV) and Slow bee paralysis virus (SBPV). Note *BQCV primers were used for nucleotide sequencing, while the same SBPV primers were used for both prevalence and sequencing.

Target	Primer name	Sequence	Amplification program	Amplicon (bp)	Reference
B. terrestris/ lucorum complex	BBM1_IGSF-1<br>BBM1_IGS_R	GGAGCAATAATTTCAATAAATAG<br>AARTTCAAAGCACTAATCTGC	15 s at 95C 15 s at 55C<br>45 s at 72C<br>x38 cycles	180<br>210	Regular Schmid-Hempel (*pers comm*)
BQCV<br>(for PCR)	BQCV4119<br>BQCV5476	TCCyCCAGTTCAACCATCTA<br>AACGTTGCCTAGrTTCGTCA	15 s at 95C,<br>30 s at 60C,<br>60 s at 72C<br>x40 cycles	1257	Lena Wilfert (*pers comm*)
*BQCV<br>(for sequencing)	BQCVLF_6527<br>BQCVLR_7513	GCGKGCCAAAGAGAGTAAGG<br>TTGyTGTTCAGTCCCCGAAT	Touchdown PCR 62C<br>15 s at 95C,<br>30 s at 62C,<br>60 s at 72C<br>X10 cycles<br><br>15 s at 95C,<br>30 s at 602 →52C,<br>60 s at 72C<br>X30 cycles	986	Lena Wilfert (*pers comm*)
SBPV	SBPV_F3168<br>SBPV_B4193	ATGCGTGATGGTCATATTCC<br>CGGTCGCTTGGTGAAAGTAT	Touchdown PCR 60C<br>15 s at 95C,<br>30 s at 60C,<br>60 s at 72C<br>x10 cycles<br><br>15 s at 95C,<br>30 s at 60 →50C,<br>60 s at 72C<br>x30 cycles	955	Lena Wilfert (*pers comm*)

Table S3. Primers and protocols used for qPCR amplification and to create plasmids for standard curves used in qPCR assays.

Primer	Sequence	Amplification program	Amplicon (bp)	Reference
SBPV_F3177<br>SBPV_B3363	GCGCTTTAGTTCAATTGCC<br>ATTATAGGACGTGAAAATATAC	10 s at 95C,<br>30 s at 60C,<br>X40 cycles<br><br>Melting curve profile:<br>55 – 95C (0.5C per second increments)	186	de Miranda *et al.* (2010)
BQCV_F7893<br>BQCV_B8150	AGTGGCGGAGATGTATGC<br>GGAGGTGAAGTGGCTATATC		257	Locke *et al.* (2012)
Rp49_qF<br><br>Rp49_qB	AAGTTCATTCGTCACCAGAG<br><br>CTTCCAGTTCCTTGACATTATG			de Miranda and Fries (2008)

Table S4. BQCV and SBPV alignment details: * note, 7 *B. lucorum* were also sequenced for SBPV but removed from the BQCV alignment because of small sample size

Virus	Genbank	Length	N total seqs	N *A.mellifera* sequences	N. *B.terrestris* sequences	N. *B pascuorum* sequences
BQCV	NC0003784.1	432	69	52	16	1
SBPV *	GU938761.1	535	78	20	19	32

Table S5. Substitution models for BQCV and SBPV alignments based on jModelTest (Guindon & Gascuel 2003; Darriba *et al.* 2012). Results of path sampling maximum likelihood estimator analysis comparing demographic and molecular clock models run in Beast 1.8. Analyses of each model in Tracer showed that a strict molecular clock could be excluded from both models; there was no significant exponential growth. Bold text highlights the chosen model for each alignment.

Fragment	Substitution model	Molecular clock	**Population demography**		
			Constant	Exponential	GMRFSkyride
BQCV	HKY + I + G	Strict	-1744.59	-1744.02	-1829.75
		Exponential	**-1741.92**	-1739.38	-1810.10
		Lognormal	-1744.27	-1740.74	-1801.56
SBPV	HKY + G	Strict	-1790.59	-1793.56	-1913.68
		Exponential	-1780.61	-1774.47	-1847.92
		Lognormal	**-1774.55**	-1775.58	-1787.82

Table S6. List of reference virus genomes used in BWA to align SMRT sequence reads. * DWV-C sequences can be found at http://www.ebi.ac.uk/ena/data/view/CEND01000001

Virus	Full virus name	Genbank Reference
BQCV	Black queen cell virus	NC-0003784
SBPV Harpenden	Slow bee paralysis virus	GU93876
SBPV Roththamsted	Slow bee paralysis virus	EU035616
DWV-A	Deformed wing virus A	NC-004830
DWV-B	Deformed wing virus B	NC-006494
DWV-C	Deformed wing virus C	ENA: CEN01000001*
SBV	Sacbrood virus	AF092924
ABPV	Acute bee paralysis virus	NC-002548
KBV	Kashmir bee virus	NC-004807
IAPV	Israeli acute paralysis virus	KY243933
CBPV	Chronic bee paralysis virus	NC-010711 and NC010712
LSv 1	Lake Sinai virus 1	HQ871931
LsV 2	Lake Sinai virus 2	HQ888865
Alpv	Aphid lethal paralysis virus	KJ817182
AmFv	Apis mellifera filamentous virus	NC- 027925
**New bumblebee viruses (Pascall *et al.* 2018)** Acry1 Bloom1 Bloom2 Bloom3 Bou1 Bou2 Bou3 Corn1 Dia1 Dicist_Full Dicist_Half Grange1 I1 I2 Mut1	N/A	N/A

N1<br>N2<br>Sac1<br>Toti1<br>Toti2<br>Toti3<br>Toti4<br>Wuchang1		

Table S7. Heat map of proportion (%) of number of PacBio sequencing reads for each virus out of the total read number. Only viruses that were present in more than one population and > 5% of the total number of reads are displayed.

Host	Site	*Varroa*	N reads	Virus SR	DWV A	DWV B	Amfv	BQCV	SBPV (Harp)	SBPV (Roth)	SBV
A.mel	Liv	y	12109	7	0.10	69.14	0.50	0.55	2.36	19.94	7.39
B.ter	Liv	y	2730	8	0.00	58.79	9.45	0.73	8.28	11.72	7.44
A.mel	IOM	n	2280	10	0.04	33.55	51.89	1.45	3.42	5.88	3.38
B.ter	IOM	n	347	9	0.29	64.55	7.49	1.15	7.20	10.37	8.07
A.mel	Le Con	y	8704	7	0.03	99.17	0.60	0.00	0.00	0.00	0.00
B.ter	Le Con	y	147	3	0.68	97.96	0.68	0.00	0.00	0.00	0.00
A.mel	Ush	n	430	4	0.00	53.49	45.81	0.23	0.00	0.00	0.00
B.ter	Ush	n	642	4	0.00	91.59	3.43	0.00	0.00	0.00	0.00

Table S8. Predicted proportions (%) of pathogen prevalence for three bee species when *Varroa* is present and absent (conversion of GLMM (table 2) estimates on the logit scale to proportions using formula exp(x)/(1+exp(x)), where x equals the parameter estimate).

Response	Species	Prevalence when *Varroa* absent	Prevalence when *Varroa* present
BQCV	*A. mellifera*	6.78	60.61
	B. pascuorum	0.064	1.34
	B. terrestris	0.45	8.74
SBPV	All species	0.04	5.95

Table S9. Bayes factors a) support for BQCV transmission between hosts b) support for BQCV transmission between locations

a)

donor	receiver Am	Bt	Bp
Am	NA	ns	7.91
Bt	ns	NA	4.94
Bp	ns	ns	NA

b)

donor	receiver IOM	Liv	Fal	Jer	Guern	Cher	Brest	Ush	Bell	Qui
IOM	NA	250	72	ns	73	ns	1559	173	287	78
Liverpool	ns	NA	ns	ns	273	ns	ns	190	ns	333
Falmouth	283	83	NA	ns	160	348	2936	ns	301	81
Jersey	ns	ns	ns	NA	349	1150	39	72	ns	ns
Guernsey	152	71	84	69	NA	5804	ns	ns	116	120
Cherbourg	ns	ns	137	195	141	NA	ns	ns	130	ns
Brest	461	175	573	1013	ns	ns	NA	ns	75	77
Ushant	ns	591	ns	53	85	ns	544	NA	94	49
Belle Ile	129	ns	ns	101	49	ns	44	ns	NA	79
Quiberon	1544	598	69	ns	131	ns	172	87	143	NA

Table S10. Bayes factors SBPV a) support for SBPV transmission between hosts b) support for SBPV transmission between locations

a)

donor	receiver Am	Bt	Bl	Bp
Am	NA	9.89	ns	30.14
Bt	9.8456	NA	9.937	11.33
Bl	ns	6.95	NA	
Bp	28.78	10.705		NA

b)

donor	recipient Cherbourg	Jersey	Guernsey	IOM	Liverpool
Cherbourg	NA	61.15	39.69	ns	ns
Jersey	23.5	NA	19.72	21.54	42.17
Guernsey	27.25	no support	NA	23.54	31.24
IOM	ns	771.82	55.62	NA	ns
Liverpool	ns	ns	37.22	ns	NA

Supplementary figures

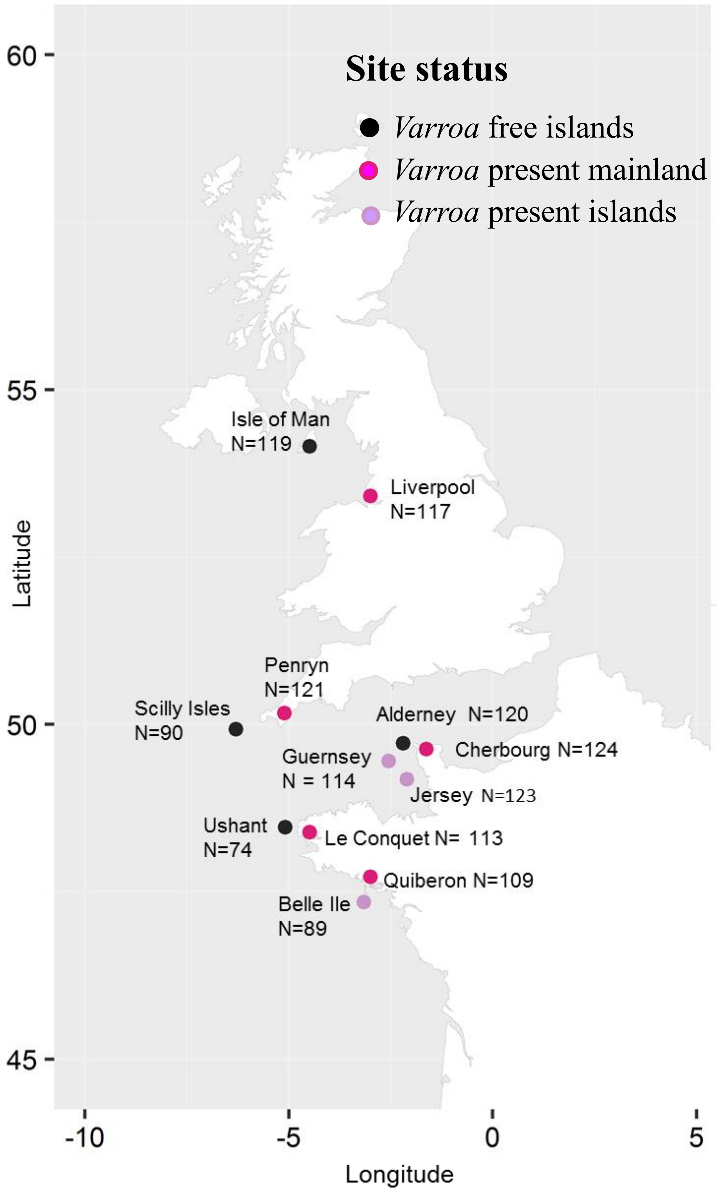

Figure S1. Sampling locations and sample sizes with *Varroa*-status shown by coloured circles.

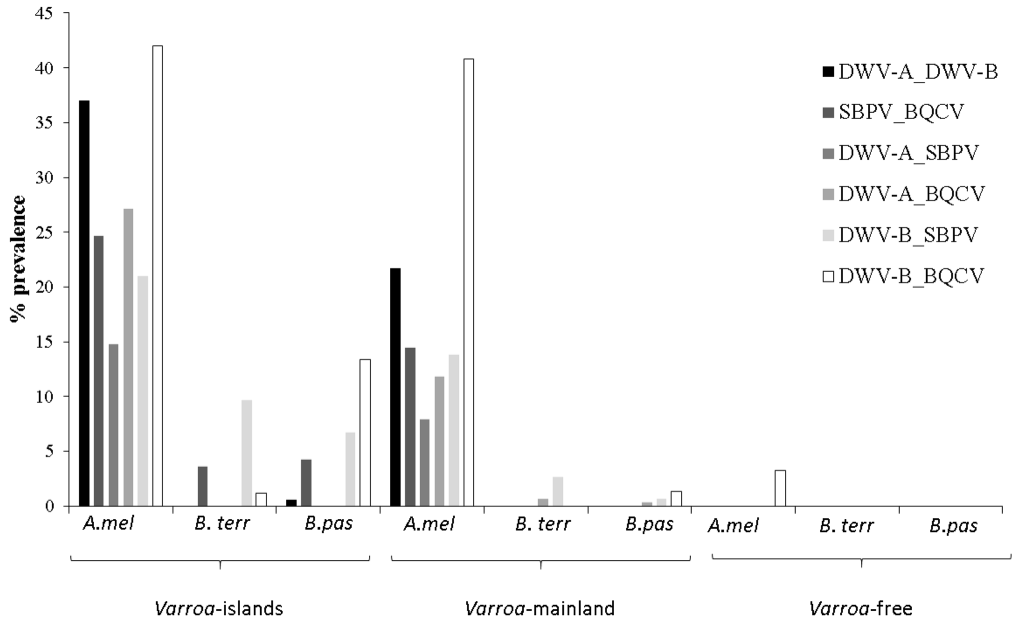

Figure S2. % prevalence of co-infections of four RNA viruses (DWV-A, DWV-B, SBPV and BQCV) by host species and *Varroa*-presence/absence (V+I = *Varroa*-present island, V+M = *Varroa*-present mainland, V- = *Varroa*-free islands.

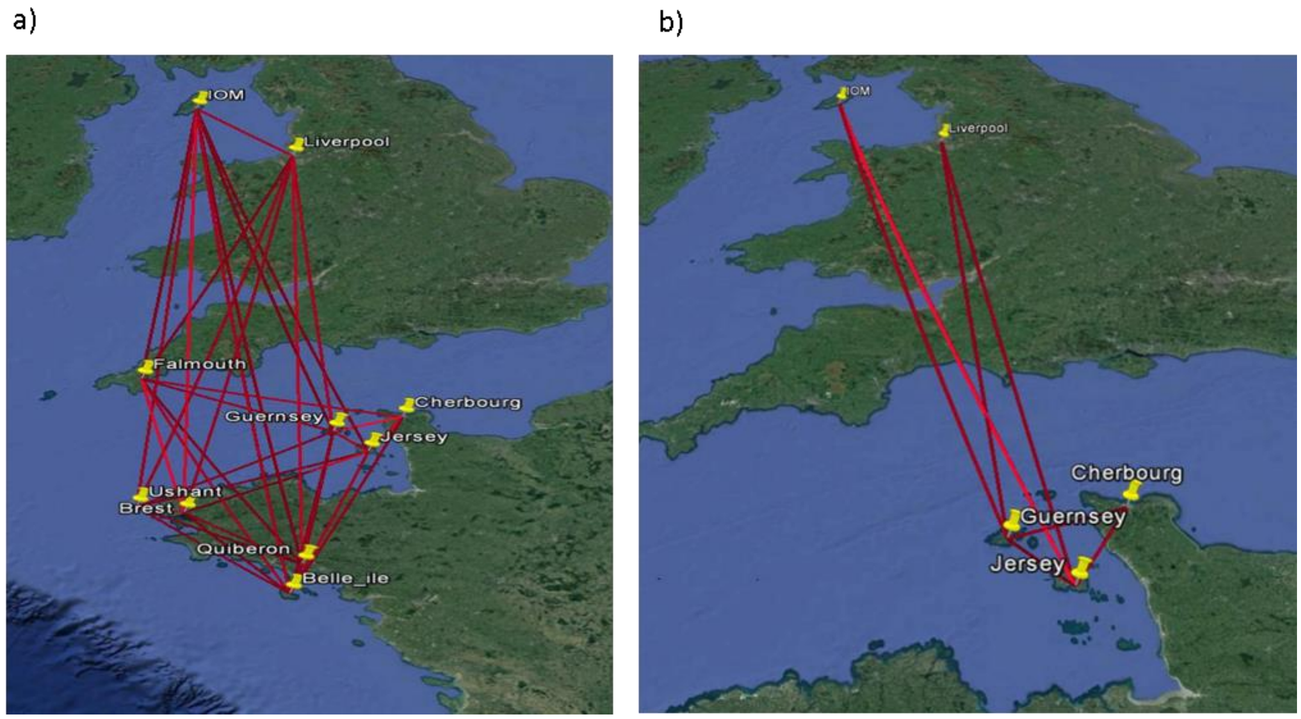

Figure S3. Map showing Bayes Factor support for BQCV (a) and SBPV (b) transmission routes between sampling locations, from Beast phylogenetic analysi

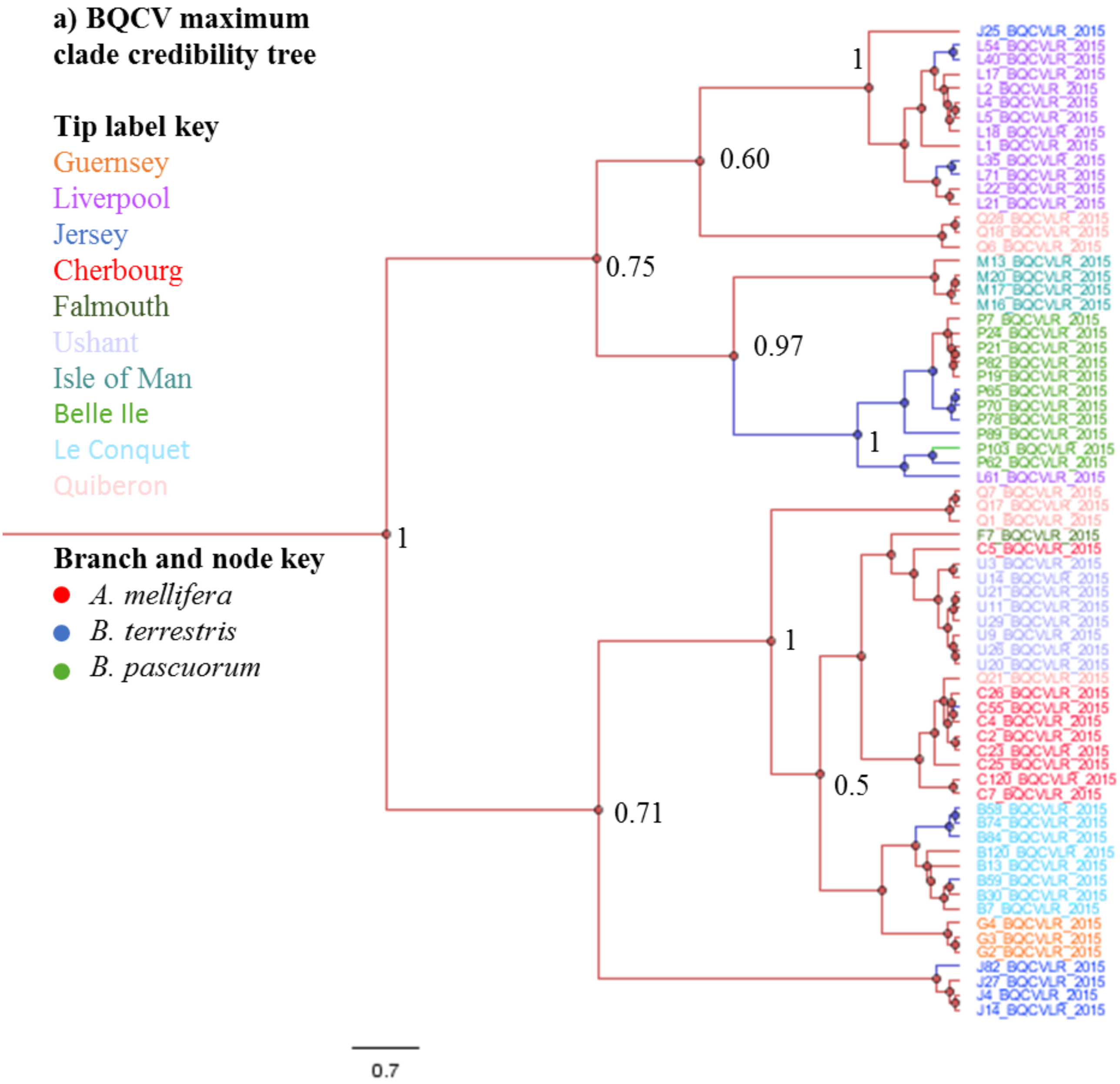

a) BQCV maximum clade credibility tree
Tip label key
Guernsey
Liverpool
Jersey
Cherbourg
Falmouth
Ushant
Isle of Man
Belle Ile
Le Conquet
Quiberon
Branch and node key
A. mellifera
B. terrestris
B. pascuorum
1
0.60
0.75
0.97
1
1
1
0.5
0.71
J25_BQCVLR_2015
L54_BQCVLR_2015
L40_BQCVLR_2015
L17_BQCVLR_2015
L2_BQCVLR_2015
L4_BQCVLR_2015
L5_BQCVLR_2015
L18_BQCVLR_2015
L1_BQCVLR_2015
L35_BQCVLR_2015
L71_BQCVLR_2015
L22_BQCVLR_2015
L21_BQCVLR_2015
Q28_BQCVLR_2015
Q18_BQCVLR_2015
Q6_BQCVLR_2015
M13_BQCVLR_2015
M20_BQCVLR_2015
M17_BQCVLR_2015
M16_BQCVLR_2015
P7_BQCVLR_2015
P24_BQCVLR_2015
P21_BQCVLR_2015
P82_BQCVLR_2015
P19_BQCVLR_2015
P65_BQCVLR_2015
P70_BQCVLR_2015
P78_BQCVLR_2015
P89_BQCVLR_2015
P103_BQCVLR_2015
P62_BQCVLR_2015
L61_BQCVLR_2015
Q7_BQCVLR_2015
Q17_BQCVLR_2015
Q1_BQCVLR_2015
F7_BQCVLR_2015
C5_BQCVLR_2015
U3_BQCVLR_2015
U14_BQCVLR_2015
U21_BQCVLR_2015
U11_BQCVLR_2015
U29_BQCVLR_2015
U9_BQCVLR_2015
U26_BQCVLR_2015
U20_BQCVLR_2015
Q21_BQCVLR_2015
C26_BQCVLR_2015
C55_BQCVLR_2015
C4_BQCVLR_2015
C2_BQCVLR_2015
C23_BQCVLR_2015
C25_BQCVLR_2015
C120_BQCVLR_2015
C7_BQCVLR_2015
B58_BQCVLR_2015
B74_BQCVLR_2015
B84_BQCVLR_2015
B120_BQCVLR_2015
B13_BQCVLR_2015
B59_BQCVLR_2015
B30_BQCVLR_2015
B7_BQCVLR_2015
G4_BQCVLR_2015
G3_BQCVLR_2015
G2_BQCVLR_2015
J82_BQCVLR_2015
J27_BQCVLR_2015
J4_BQCVLR_2015
J14_BQCVLR_2015
0.7

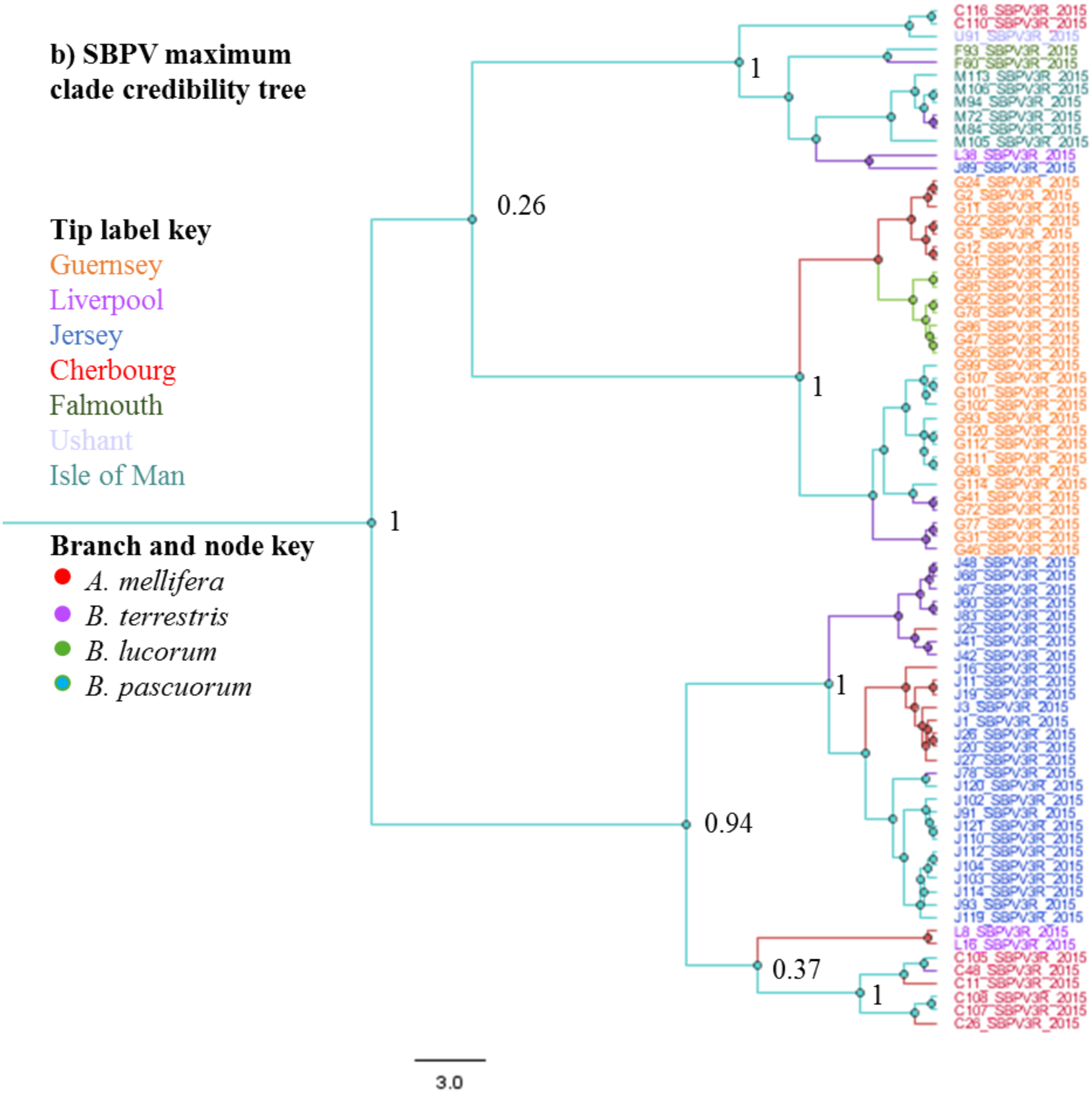

Figure S4. Maximum clade credibility (MCC) tree for BQCV (69 sequences of a 432nt fragment) (a) and SBPV (78 sequences of a 535nt fragment) (b) showing host and location structure. Branches and nodes are coloured according to host species and the branch tips are coloured by location (see key). Posterior support is indicated for nodes up to the 3rd order. The scale is time, in years.

References

Darriba, D., Taboada, G.L., Doallo, R. & Posada, D. (2012). jModelTest 2: more models, new heuristics and parallel computing. *Nature Methods*, 9, 772 DOI: https://doi.org/10.1038/nmeth.2109.

de Miranda, J.R., Dainat, B., Locke, B., Cordoni, G., Berthoud, H., Gauthier, L. *et al.* (2010). Genetic characterization of Slow Bee Paralysis Virus of the honeybee (*Apis mellifera* L.). *Journal of General Virology*, 91, 2524-2530.

de Miranda, J.R. & Fries, I. (2008). Venereal and vertical transmission of Deformed Wing Virus in honeybees (*Apis mellifera* L.). *Journal of Invertebrate Pathology*, 98, 184-189.

Guindon, S. & Gascuel, O. (2003). A simple, fast and accurate method to estimate large phylogenies by maximum-likelihood. *Systematic Biology*, 52, 696-704.

Locke, B., Forsgren, E., Fries, I. & De Miranda, J.R. (2012). Acaricide tratment affects viral dynamics in *Varroa destructor*-infested honey bee colonies via both host physiology and mite control. *Applied and Environmental Microbiology*, 78, 227-235.

Pascall, D.J., Tinsley, M.C., Obbard, D., J, & Wilfert, L. (2018). Host evolutionary history predicts virus prevalence across bumblebee species *BioRxiv* DOI: https://doi.org/10.1101/498717.
